# Therapeutic management of hypophosphatemic rickets from infancy to adulthood

**DOI:** 10.1530/EC-13-0103

**Published:** 2014-03-15

**Authors:** Agnès Linglart, Martin Biosse-Duplan, Karine Briot, Catherine Chaussain, Laure Esterle, Séverine Guillaume-Czitrom, Peter Kamenicky, Jerome Nevoux, Dominique Prié, Anya Rothenbuhler, Philippe Wicart, Pol Harvengt

**Affiliations:** 1 Service d'Endocrinologie et Diabétologie de l'Enfant Hôpital Bicêtre, APHP 78 rue du Général Leclerc , Le Kremlin Bicêtre, 94270 France; 2 Service de Pédiatrie générale – Consultation de rhumatologie Hôpital Bicêtre, APHP 78 rue du Général Leclerc , Le Kremlin Bicêtre, 94270 France; 3 Service d'Endocrinologie et des Maladies de la Reproduction Hôpital Bicêtre, APHP 78 rue du Général Leclerc , Le Kremlin Bicêtre, 94270 France; 4 Service d'ORL et chirurgie cervico-maxillo-faciale Hôpital Bicêtre, APHP 78 rue du Général Leclerc , Le Kremlin Bicêtre, 94270 France; 5 Université Paris 11 faculté de Médecine, Hôpital Bicêtre 70 rue du général Leclerc, Le Kremlin-Bicêtre, 94270 France; 6 Centre de Référence des Maladies Rares du Métabolisme du Calcium et du Phosphore Le Kremlin-Bicêtre France; 7 Service d'Odontologie-Maladies Rares Hôpital Bretonneau 2 rue Carpeaux Paris, 75018 France; 8 Université Paris Descartes 12 Rue de l'École de Médecine Paris, 75006 France; 9 Service Rhumatologie B Hôpital Cochin, APHP 27, rue du Faubourg Saint-Jacques, Paris, 75014 France; 10 Centre de Référence des Maladies Rares des Maladies Auto-Inflammatoires Rares de l'Enfant Le Kremlin Bicêtre France; 11 Service d'explorations fonctionnelles rénales, Hôpital Necker-Enfants Malades 149 rue de Sèvres, Paris, 75015 France; 12 Service de Chirurgie infantile orthopédique Hôpital Necker-Enfants Malades 149 rue de Sèvres, Paris, 75015 France; 13 Association de patients RVRH-XLH 20 rue Merlin de Thionville, Suresnes , 92150 France

**Keywords:** calcium, bone, rare diseases/syndromes, X-linked hypophosphatemic rickets

## Abstract

In children, hypophosphatemic rickets (HR) is revealed by delayed walking, waddling gait, leg bowing, enlarged cartilages, bone pain, craniostenosis, spontaneous dental abscesses, and growth failure. If undiagnosed during childhood, patients with hypophosphatemia present with bone and/or joint pain, fractures, mineralization defects such as osteomalacia, entesopathy, severe dental anomalies, hearing loss, and fatigue. Healing rickets is the initial endpoint of treatment in children. Therapy aims at counteracting consequences of *FGF23* excess, i.e. oral phosphorus supplementation with multiple daily intakes to compensate for renal phosphate wasting and active vitamin D analogs (alfacalcidol or calcitriol) to counter the 1,25-diOH-vitamin D deficiency. Corrective surgeries for residual leg bowing at the end of growth are occasionally performed. In absence of consensus regarding indications of the treatment in adults, it is generally accepted that medical treatment should be reinitiated (or maintained) in symptomatic patients to reduce pain, which may be due to bone microfractures and/or osteomalacia. In addition to the conventional treatment, optimal care of symptomatic patients requires pharmacological and non-pharmacological management of pain and joint stiffness, through appropriated rehabilitation. Much attention should be given to the dental and periodontal manifestations of HR. Besides vitamin D analogs and phosphate supplements that improve tooth mineralization, rigorous oral hygiene, active endodontic treatment of root abscesses and preventive protection of teeth surfaces are recommended. Current outcomes of this therapy are still not optimal, and therapies targeting the pathophysiology of the disease, i.e. FGF23 excess, are desirable. In this review, medical, dental, surgical, and contributions of various expertises to the treatment of HR are described, with an effort to highlight the importance of coordinated care.

## Introduction

Phosphate wasting ineluctably leads to hypophosphatemia and numerous consequences including mineralization defects. In children, hypophosphatemia is revealed by vitamin D-resistant rickets and results in variable degrees of delayed walking, waddling gait, leg bowing, enlarged cartilages, bone pain, craniostenosis, spontaneous dental abscesses, and growth failure. If undiagnosed during childhood, hypophosphatemia is suspected when patients present with bone and/or joint pain, fractures, mineralization defects such as osteomalacia, entesopathy, severe dental anomalies, hearing loss, and fatigue. Symptoms might be present, although to a lesser degree, in adults who underwent the conventional treatment throughout their childhood and adolescence. Causes of phosphate wasting are mostly due to genetic defects in factors necessary for phosphate handling; for a review read (1). They have been summarized in Table 1. In this review, we will consider two different types of phosphate wasting. Firstly, phosphate wasting may be secondary to increased fibroblast growth factor 23 (FGF23) signaling, which is a circulating factor secreted by osteoblasts, odontoblasts, and osteocytes (2). In the renal proximal tubule, FGF23 inhibits the sodium-phosphate transport through ion channels, NPT2a and NPT2c, and prevents 1,25-diOH-vitamin D production. As a consequence, both renal and digestive absorption of phosphate are diminished. Recently, extra renal effects of FGF23 have been reported, such as immune function in human monocytes (3), iron (4, 5), glucose (6) and lipid metabolism (7, 8).

Causes of phosphate wasting secondary to elevated FGF23 mainly encompass not only X-linked hypophosphatemic rickets (XLHR) due to loss-of-function mutations in *PHEX*, an endopeptidase encoded by a gene localized on the X chromosome (9), but also autosomal dominant hypophosphatemic rickets (ADHR) due to recurrent heterozygous mutations affecting the ^176^RXXR^179^ motif in FGF23 (10), autosomal recessive HR (ARHR) due to loss-of-function mutations in Dentin matrix protein 1 (*DMP1*) (11) or *ENPP1* (12), and uncontrolled secretion of FGF23 by mesenchymal tumors, a condition known as tumor-induced osteomalacia (TIO) (13). Except for the latter, mineralization defects involving bone and teeth are caused by hypophosphatemia and FGF23 excess and, in addition, by a direct effect of the absence of functional *PHEX* (or *DMP1*) on bone or tooth extracellular matrix (ECM) mineralization (14).

Secondly, phosphate wasting may be due to a primary renal tubular defect, i.e. hereditary HR with hypercalciuria (HHRH) due to molecular defects of the sodium-phosphate channel NPT2c, diseases affecting several renal tubular functions such as Dent or Lowe syndromes, or tubular toxicity of drugs (15, 16). All these conditions share a diminished capacity to transport phosphate from the glomerular filtrate to the blood circulation. In response to hypophosphatemia, FGF23 secretion is adequately suppressed, and 1,25-diOH-vitamin D production and absorption of calcium through the gut and urinary calcium excretion are consequently enhanced.

Besides acquired disorders like tumors or drug toxicity, most conditions leading to phosphate wasting are congenital and will continue throughout the patient's lifetime. To this day, therapy has mostly been evaluated in children. Enormous progress has been made since the availability of vitamin D analogs, such as calcitriol and alfacalcidol, in the mid-1970s and the evolution of surgical procedures. However, two major issues remain: i) the growth retardation and recurrent dental infections in children and ii) the necessity of adequate therapeutic strategies in adults. Indeed, the disease remains physically apparent and the global objective of therapies (medical, dental, and surgical) should be to limit, and in best cases avoid, sequel by correcting leg deformities, promoting growth and preserving dentition. In this context, we will describe in this review the current and future treatments available to counteract phosphate wasting, restore serum phosphate and allow adequate bone and tooth mineralization.

## Therapy in children

### Medical treatment for phosphate wasting

**Figure 1:**
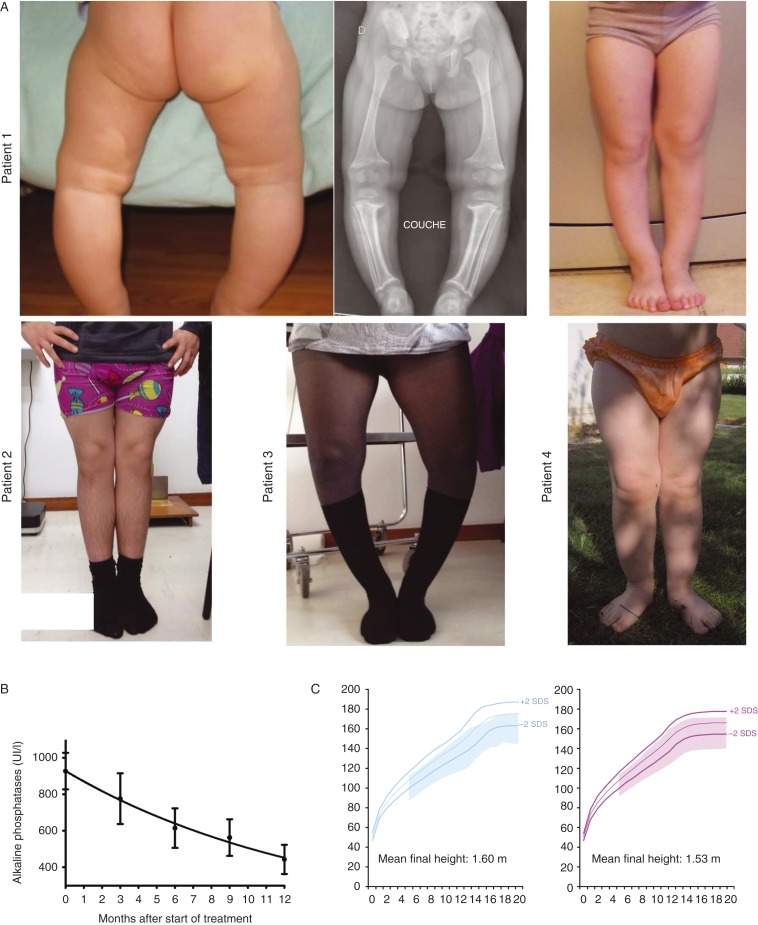
Evolution of clinical (leg bowing and growth) and biochemical parameters (alkaline phosphatase levels) during treatment with vitamin D analogs and phosphate supplements in children. (A) Patient 1 is a 2-year-old girl (left an middle panels) recently diagnosed with XLHR and a *de novo* mutation of *PHEX*. The same girl is shown at the age of 5 years with straight legs (right panel). Patient 2 is a 14-year-old boy who was treated since the age of four (XLHR and a *de novo* mutation of *PHEX*). Patient 3 is a 13-year-old girl who presents with persistent leg bowing despite being treated since she was 3 years old (XLHR and a *de novo* mutation of *PHEX*). Patient 4 is a 2-year-old girl who started therapy at the age of 4 months. Diagnosis of XLHR was made in the context of familial disease (mother and two sisters affected). (B) Evolution of alkaline phosphatase levels throughout the first year of therapy in 30 patients affected with HR and elevated FGF23. (C) Growth pattern (range between +2 and −2 SDS is shadowed) in 32 girls and 29 boys affected with HR and elevated FGF23 and receiving vitamin D analogs and phosphate supplements throughout childhood and puberty. Mean, +2, and −2 SDS of French reference growth charts are represented by colored lines.

Healing rickets by normalizing serum alkaline phosphatase (ALP) levels and radiological signs is the initial endpoint in children. Treating rickets will promote growth, progressively correct leg deformities (Fig. 1A), and facilitate tooth mineralization. In infants diagnosed before they even show signs of rickets, the treatment goal for them will be not to develop rickets (Fig. 1A). Earlier treatment has been shown to lead to better results (17). Objectives, which could also be described as expected results, are described in Table 2. The treatment intends to, in the following order: i) reduce bone pain, ii) normalize (or near-normalize) ALP levels (Fig. 1B), iii) improve growth (Fig. 1C), and iv) restore straight legs and improve teeth health.

Nowadays, the medical treatment is aimed at counteracting consequences of FGF23 excess, i.e. oral phosphorus supplementation with multiple daily intakes to compensate for renal phosphate wasting and active vitamin D analogs (alfacalcidol or calcitriol) to counter the 1,25-diOH-vitamin D deficiency (Table 3). The daily doses of phosphorus supplements range between 40 (adolescents) and 60 (toddlers) mg/kg per day (Table 4). Multiple daily doses of phosphate supplements are mandatory throughout childhood and adolescence, because, in the context of diminished phosphate reabsorption, serum phosphate level is back to low baseline few hours after phosphate intake. At these doses, digestive complaints are extremely rare. The daily dose of phosphate supplements is adjusted to efficacy (i.e., ALP levels, leg bowing, and growth velocity), patient's weight, and PTH levels. Note that serum phosphate is not used to adjust phosphate therapy. On the contrary, as fasting phosphate is not restored by treatment, increasing phosphate supplement doses leads to intestinal discomfort and secondary (sometimes tertiary) hyperparathyroidism. Urinary phosphate measured on a 24-h collection should parallel the daily intake of phosphate supplements, and may be used to check for compliance. Prescribing phosphate intake is a balance between excessive dosage tending to hyperparathyroidism and insufficient dosage slowing the healing of rickets. 1α-hydroxylated vitamin D analogs constitute the second pillar of conventional treatment. Their half-life is sufficient to allow for single and twice daily oral doses of alfacalcidol and calcitriol respectively. The starting dose of vitamin D analogs (usually 1–2 μg/day alfacalcidol or 0.5–1 μg/day calcitriol) depends on growth velocity. Higher doses (1–3 μg/day alfacalcidol or 0.5–1.5 μg/day calcitriol) are associated with periods of high growth velocity, such as early childhood and adolescence. Vitamin D analog doses are adjusted to give the maximum dose for efficacy based on ALP levels, leg bowing, and growth velocity, without reaching toxicity, mainly hypercalciuria. Hypercalciuria does not usually occur until ALP levels are normalized. As it is not invasive to collect urine, we recommend measuring urinary calcium and creatinine every 3 months on a spot urine collection in young children and on a 24-h collection in older children (>5) toilet trained. Hypercalciuria is defined by urinary calcium/urinary creatinine above 1 mmol/mmol (0.35 mg/mg) in toddlers and 24-h urinary calcium excretion above 5 mg/kg per day in older children. We recommend screening for nephrocalcinosis with yearly ultrasound (every second year in the absence of episodes of hypercalciuria). It is common, after healing rickets, for initial dosage of vitamin D analogs to be decreased to maintenance doses.

As for many chronic diseases, compliance to oral treatment is a major issue, even in expert hands. For optimal dosage, it is difficult to base our guidance on doses reported in published studies, which are all over 20 years old and report a very wide range of doses between 10 and 80 ng/kg per day of calcitriol and 30 and 180 mg/kg per day of elemental phosphorus (18, 19, 20, 21, 22, 23, 24). Our recommendations are based on over 30 years of experience at our center in treating over 250 patients with HR and are similar to the guidelines advised recently by Carpenter *et al*. (25). In addition, we prescribe 25-OH-vitamin D supplements and optimize dietary calcium intake in children, although no published studies support this point.

Healing active rickets promotes growth and after 2 years of successful treatment, patients' growth velocity is restored to its maximal potential in a majority of patients. However, 25–40% of patients with well-controlled XLHR show linear growth failure despite optimal treatment and have a final height under −2 SDS (19, 21, 26, 27, 28, 29, 30, 31, 32, 33) (and our experience, Fig. 1C). Until recently, only limited pilot studies (small patient numbers, limited period of observation, lack of controls and randomizations) have been conducted, which suggest a beneficial effect of recombinant growth hormone (rGH) treatment on growth velocity in patients with XLHR (29, 34, 35, 36, 37, 38). The only randomized study by Zivicnjak *et al*. (39) showed significant improvement of linear growth (+1.1 height SDS) in eight patients treated with rGH out of 16 short (mean height SDS −3.3) prepubertal children with XLHR. Addition of rGH induces a rise in mineral needs, which should be accompanied with a 20–30% increment in vitamin D analogs dosage. Only well-observant patients with healed rickets can benefit from rGH.

In patients with HHRH, 1,25-diOH-vitamin D synthesis is enhanced and PTH secretion often suppressed, both conditions favoring hypercalciuria (40); therefore, patients require treatment with phosphate supplements solely. Doses and adjustment rules are similar to that used for XLHR patients. In this condition, 25-OH-vitamin D supplementation should be monitored very carefully by trained physicians to avoid increase in 1,25-diOH-vitamin D generation and hypercalciuria.

### Orthopedic and surgical management in children

Hypophosphatemia induces progressive bowing of the legs that becomes apparent with the onset of weight bearing (Fig. 1A), and may hinder the walking capacity. These mal-alignments are characterized by diaphyseal–metaphyseal regions of lower limbs long bones bowing in frontal plan with varus or valgus combined with a degree of flexion. Internal torsion of the tibia and fibula is frequent, as well as anteverted femoral neck. As a consequence, patients report lower limb pain; they also may develop patellar dysplasia including chondromalacia, lateral femoro-patellar subluxation, and gait troubles. Usually, the response of pain and skeletal deformities to medical treatment with adequate doses of calcitriol and phosphorus ± class I analgesics (acetaminophen, paracetamol, some NSAIDs) is good, but bowing may not be entirely resolved and moderate bone pain may persist. Physiotherapy can be useful at this stage to prevent complete patellar dislocation and improve muscle fitness; sitting on the feet should be prohibited in children to decrease femoral neck anteversion. A disproportionate muscular insufficiency was described, but the relative responsibility of hypophosphatemia versus lower limb bowing – which modifies the muscular work – was not evaluated (41). Most of our patients show joint hyperlaxity and increased skin elasticity. Physiotherapy to improve joint stability with muscle reinforcement can be offered to patients.

**Figure 2:**
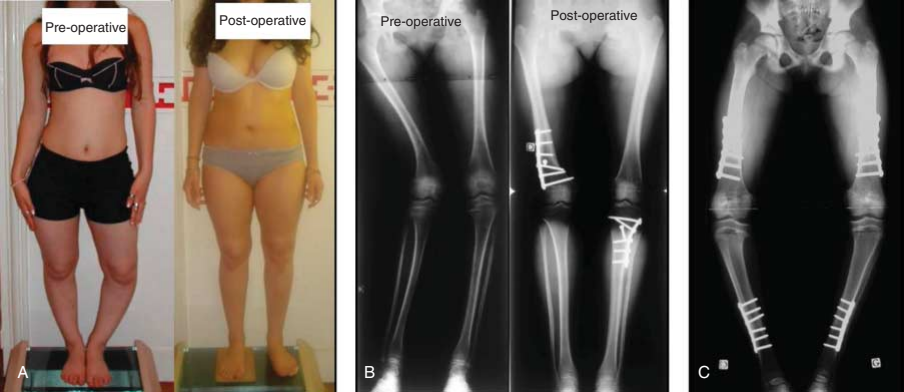
Surgeries in children and adolescents with XLHR. (A) Bilateral lower limbs mal-alignment including distal right femur valgus and proximal left tibia varus – pre- and post-operative aspects (distal right femur varization and proximal left tibia valgization osteotomies). (B) Pre- and post-operative radiological aspect of the patient displayed in (A). (C) Eight-year-old girl with XLHR and severe pre-operative deformations impeding joints mobility. Recurrence of leg bowing after surgery likely due to compliance issues to phosphate and vitamin D analogs. Bilateral medial proximal tibiae epiphysiodesis inducing varus deformations.

When leg bowing persists despite ‘optimal’ treatment, bone distortions should be assessed through the low-irradiating EOS system, which provides a 3D reconstruction of lower limbs bones in standing position. It may be combined with CT scanning to measure the degree of torsion. Surgery during childhood should be avoided. Because of open epiphyses, patients present a significant risk of recurrence of the bowing at the level of osteotomy or secondary to the adjacent epiphysiodesis (Fig. 2). When necessary, due to major bone deformities, surgery should be combined with adjusted doses of phosphate supplements and vitamin D analogs in order to prevent recurrence as previously evoked.

The actual place of the surgery is the correction of residual deformities at the end of growth (Fig. 2). The achievement of horizontal knee joints often requires bifocal femoral and tibiae metaphyseal–diaphyseal osteotomies. Osteosynthesis is done by locking plates or intramedullary nails (42). Progressive correction using external fixation is an alternative that provides precise 3D angular deformities management (43). Distal tibiae varus with significant ankle joints obliquity should be operated upon with supramalleolar dome osteotomy (44). Fusion is usually acquired, without tendency of delayed fusion or pseudarthrosis. In case of significant lateral subluxation after alignment correction, surgery of the femoro-patellar joint is also required. However, ever since the implementation of the usage of vitamin D analogs, surgical indications have been considerably diminished. In our experience, recourse to surgery dropped from 89 to 11% when we started to use the modern medical treatment in patients born after 1975.

Most hypophosphatemic patients present with increased head length and frontal bossing. Craniosynostosis and sometimes Chiari malformations giving rise to headaches and vertigo may also affect patients and require neurosurgery when symptomatic.

## Therapy in adults

### Metabolism

After the years of burdensome therapy over the ‘pediatric’ period, which is essential to ensure adequate bone matrix mineralization and skeletal growth, young adult patients with hereditary hypophosphatemia (HH) often stop treatment. Lessened parental surveillance, poor taste of phosphate preparations, and lack of convincing demonstration of therapeutic benefits in asymptomatic individuals contribute to the low compliance in (young) adults. Nevertheless, the metabolic and endocrine consequences of chronic phosphate wasting persist life long. Adult endocrinologist following patients with HH thus necessarily faces two key questions: i) whom to treat and ii) what kind of treatment to give?

**Figure 3:**
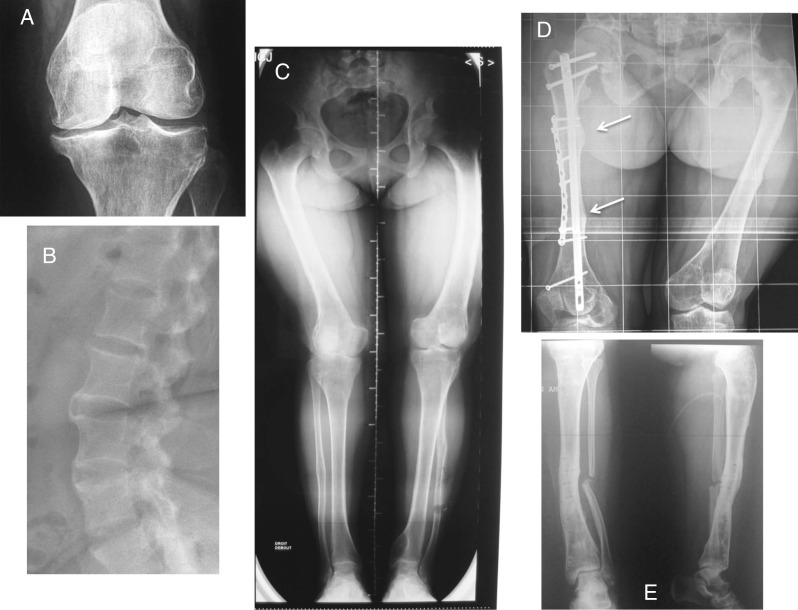
Various burdens of the disease in adults leading to resume therapy with phosphate supplements and vitamin D analogs. (A) Osteoarthritis of the knee in a 28-year-old woman with persistent bone deformities after adolescence. (B) Spinal enthesopathies of a 35-year-old patient with XLHR. (C) Lower limb deformities in a young adult requiring corrective surgery. (D) Dramatic consequences of rickets and osteomalacia in a 30-year-old patient who did not receive vitamin D analogs. Arrows show insufficiency fractures. Bone demineralization and hip osteoarthritis are visible. (E) Delayed healing of fibulae fractures in the same patient following corrective surgeries on both tibias.

There is no consensus regarding indications of the treatment in adult patients. It is generally accepted that treatment should be reinitiated (or maintained) in all symptomatic patients in order to reduce pain, which may be due to bone microfractures and/or osteomalacia (25, 45) (Fig. 3). Patients with significant reduction in pain symptoms remain the most compliant. Patients with planed surgical interventions (i.e., corrective osteotomy, dental implants) should also be temporarily treated to promote bone mineralization (25). The conventional treatment in these adult patients is based on oral phosphate salts, usually given twice daily, and active vitamin D metabolites. The aim of the treatment is to improve the symptoms, not to normalize serum phosphate levels. Careful monitoring of plasma calcium, PTH, creatinine, and 24-h urinary calcium excretion is required (25, 46) in order to prevent tertiary hyperparathyroidism, induced by phosphate overdose (47), and hypercalciuria with nephrocalcinosis and renal insufficiency, resulting from calcitriol overtreatment (48). In our experience, tertiary hyperparathyroidism in patients with XLHR is rare (A Linglart, P Kamenicky, D Prie, A Rothenbuhler, unpublished observations) and should be preferentially treated surgically, even though beneficial effects of adjunctive therapy by 24,25-dihydroxyvitamin D has also been reported in one study (49).

Considering the lack of evidence for clinical benefit and the possible side effects, indication of the conventional phosphate- and vitamin D analog-based therapy in asymptomatic patients is questionable. Long-term consequences of chronic hypophosphatemia in adult individuals are not known. Chronically decreased 1,25-diOH-vitamin D synthesis may have a significant impact on health, given the numerous beneficial effects of vitamin D on metabolism, cardiovascular system, cancer prevention, and immune functions (50). Treatment should be initiated at least in situations of increased demands on phosphate and calcium, such as pregnancy, to ensure adequate mineralization of the fetal skeleton, or lactation, to enable sufficient galactopoiesis, and in both cases to prevent worsening of the phosphate deficit in the maternal organism (51).

Conventional medical therapies of FGF23-related hypophosphatemic disorders consist in substituting the consequences of the FGF23 excess (46). Nevertheless, increased FGF23 levels may have deleterious effects on health *per se*, especially on metabolism and cardiac functions (8, 52, 53). New therapeutic approaches targeting FGF23 actions in general, including its impact on glucose and lipid metabolism, are very interesting in adult patients with HH, since they frequently present with reduced mobility and are thus prone to develop obesity and metabolic syndrome.

### Rheumatology

Although disease severity is variable, adults with HR may suffer from osteoarticular symptoms, such as pain and joint stiffness, leading to disability of physical function and poor quality of life (25). The conventional treatment with vitamin D analogs and phosphate supplements aims to decrease osteoarticular symptoms and improve physical function. However, only limited data supports the efficiency of the therapy in adults presenting these complications. Moreover, bone/joint pain may have different origin such as osteomalacia, insufficiency fractures, osteoarthritis, and enthesopathy (Fig. 3). Identifying the cause of the pain should be considered as the first step for the optimal management of osteoarticular symptoms. Osteomalacia and spontaneous insufficiency fractures, which occur in the lower extremities of the weight bearing bones, should trigger treatment in adults. In an open-label study conducted in 16 symptomatic adult patients with XLHR, Sullivan *et al*. (45) showed that the combination of calcitriol and phosphate supplements decreases bone pain, increases serum phosphate, and reduces the extent of osteomalacia quantified by pre- and post-treatment bone biopsies. Moreover, insufficiency fractures usually heal faster with this conventional treatment. Despite the mineralization defect, so-called osteomalacia, adults with XLHR present with paradoxical heterotopic ossifications of tendon and ligament insertion sites. This leads to the formation of enthesophytes in fibrocartilaginous tissues, often painful and causing joint dysfunction. Risk factors associated with the occurrence of enthesopathy are unknown; vitamin D analogs and phosphate supplements do not prevent this complication (54). Apart from symptomatic patients at increased risk of insufficiency fractures and osteomalacia, treatment has been proposed to patients with planed surgical interventions (osteotomy, joint replacement) (see above). Treatment should be also discussed in asymptomatic women at the time of menopause, in the absence of estrogen substitution, to prevent osteomalacia. In addition to the conventional treatment, optimal care of symptomatic patients requires pharmacological and non-pharmacological management of pain and joint stiffness, through appropriated rehabilitation. Individualized exercises and adapted physical activity should be proposed to improve physical function and reduce the metabolic consequences of XLHR.

Novel therapies targeting FGF23 actions (see below) are awaited to fulfill the current unmet needs, such as diminished motor function or prevention of enthesis ossification. For the latter, enthesis fibrocartilage cells of *Hyp* mice (murine homolog of XLHR) specifically express FGFR3 and Klotho, thereby suggesting that FGF23 inhibition might prevent fibrocartilage ossification (54).

## Ears

Patients with HR present inconstant and variable hearing loss depending on age and cause of phosphate wasting. Hearing impairment has been described in mouse models and human disorders due to increased FGF23 signaling, yet not with primary renal tubular defect. Hearing difficulties appear during adulthood in treated patients with XLHR (55). Patients may have mild-to-severe sensorineural hearing loss, affecting mainly low and high frequencies (56). Some patients also present with tinnitus and vertigo associated with low frequencies hearing loss similar to that of Menière's disease (57). X-rays show generalized osteosclerosis and thickening of the petrous bone, with narrowed internal auditory meatus (58). In rodents models mimicking XLHR, mice display variable expressions of deafness, circling behavior, lack of postural reflexes, and cranial dysmorphology (59, 60, 61). Differently, hearing loss has been reported in HR children with mutations in *ENPP1* – as early as 9 days of life – or *DMP1* (62, 63). Overall, hearing loss resembling stapes otosclerosis occurs early in life in ARHR patients, while hearing loss resembling Menière's disease develops after the second decade in XLHR patients. We presently do not know whether phosphate supplements and vitamin D analogs modify the hearing evolution.

## Dental and periodontal defects

**Figure 4:**
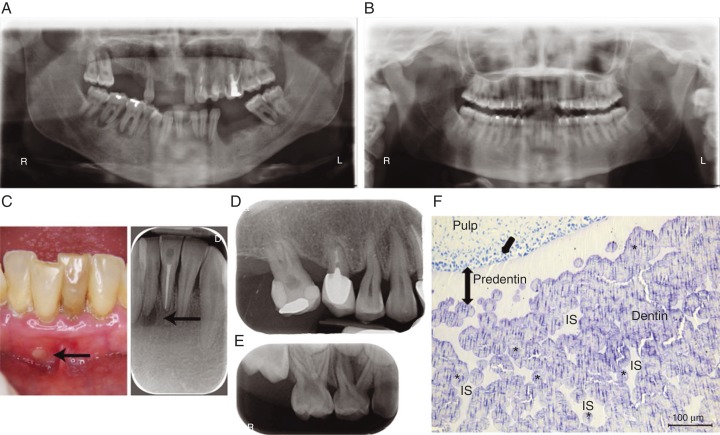
Dental defects in patients with X-linked hypophosphatemia (XLH). (A) Orthopantomogram of a 35-year-old XLH patient. Note multiple absent teeth and endodontic lesions. (B) Orthopantomogram of a 30-year-old XLH patient that benefited from vitamin D analogs and phosphate supplements during growth with good compliance. No dental or periodontal defects are evident. (C) Intraoral view and corresponding X-ray of an endodontic infection (arrows) affecting the intact central right lower incisor in a 35 year-old XLH patient. (D) Enlarged pulp chambers, prominent pulp horns, radiolucent hypomineralized dentin and endodontic infection in a 6-year-old XLH patient. (E) Alveolar bone loss in a 45-year-old XLH patient. (F) Toluidine blue-stained section of a third molar germ of a 14-year-old female with XLH showing abnormal dentin mineralization. Numerous nonmineralized interglobular spaces (IS) are observed between unmerged calcospherites in the dentin body (document laboratory EA2496, Dental school University Paris Descartes, France). Asterisks indicate calcospherites, single arrow indicates dentin secreting cells, odontoblasts, and double head arrow indicates extent of predentin.

The presence of severe dental manifestations in patients with HR resulting either from *PHEX* mutations or from other causes summarized above, including mutations in *DMP1* or *FGF23*, is now well recognized (Fig. 4). The dominant feature is the occurrence of spontaneous infection of the dental pulp tissue, resulting in tooth abscesses. In contrast with common endodontic infection, these abscesses develop in teeth without any signs of trauma or decay, affecting both the deciduous and permanent dentition (64, 65). Clinically, teeth of patients with HR look normal, complicating the identification of the causal tooth and the diagnosis of the endodontic origin of the infection. Radiographically, the enamel layer appears thinner while the dentin layer is more radiolucent. Pulp chambers are enlarged, resembling taurodontism and prominent pulp horns extend up to the dentino–enamel junction (64). Extensive enamel cracking and fissuring can be observed on histologic sections, as well as dentin mineralization defects. Unmerged dentin calcospherites are observed and are separated by large non-mineralized interglobular spaces (66, 67) (Fig. 4F). The endodontic infections are believed to result from rapid dental pulp necrosis, a consequence of the abnormal dentin mineralization and enamel cracks that allow invasion of the pulp by oral bacteria (68).

The dentin ECM is secreted by odontoblasts which, like osteoblasts, express high levels of proteins involved in mineral ion homeostasis and in the binding and proteolytic processing of other proteins and peptides regulating mineralization (69, 70). Hence, the abnormal hypophosphatemic dentin contains degraded fragments of noncollagenous phosphorylated matrix proteins including matrix extracellular phosphoglycoprotein (MEPE), DMP1, and osteopontin (OPN), and particularly peptides with the acidic serine- and aspartate-rich motif (ASARM) (71, 72). Interestingly, PHEX is the only known enzyme capable of cleaving ASARM peptides, whose accumulation leads to inhibition of mineralization.

Because the pain and swelling that result from tooth abscesses are usually detected by patients or their families and will bring them to consult, much attention has been drawn to the dental manifestation of HR. However, it is becoming clear that their periodontal health is also affected, especially in adults. Comparing the periodontal status of ten adults with familial HR with age-matched controls (73), it was observed that the prevalence of periodontitis was high in hypophosphatemic patients (60% vs 3.6% to 7.3% in controls). Our unpublished data on the periodontal status of more than 20 consecutive adult patients with HR revealed an increased prevalence and severity of periodontitis when compared with age-matched controls, despite a similar gingival inflammation (Fig. 3A, D and E). Although, no published studies have explored non-surgical and surgical periodontal treatments in adults with low phosphate, we observed a favorable response to these treatments. The importance of the supportive periodontal therapy in these patients cannot be overlooked. One case of implant placement along with guided bone regeneration in a XLH adult patient was reported with satisfactory outcomes after 42 months (74). Consistent with constitutional defects of periodontal tissues, mice lacking *Dmp1* or *Phex* have a defective alveolar bone and cementum (75, 76).

**Figure 5:**
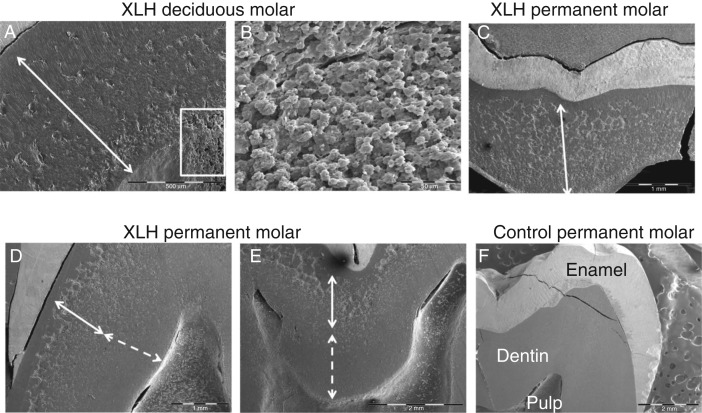
Scanning electron microscopy views (documents laboratory EA2496, Dental school University Paris Descartes, France) of (A) A deciduous molar of a 6-year-old male patient with XLHR, born from a XLH mother, showing that the major bulk of dentin (arrow) is abnormally mineralized. (B) At higher magnification (white rectangle), the dentin appears extremely porous with multiples unmineralized spaces. (C) Permanent molar of an adult XLH patient hich was not treated during growth. All the dentin bulk (arrow) is abnormally mineralized. (D and E). Permanent third molar of a 15-year old male patient with XLH who was treated during growth with a good compliance to therapy but a late onset. Remaining calcospherites are observed in the outer part of dentin (full arrow) corresponding to infancy, whereas good mineralization (dotted arrow) is seen in the inner part of dentin corresponding to the treatment period. (F) Control permanent molar.

Conventional therapy with vitamin D analogs and phosphate supplements has a substantial beneficial impact on oral health that will depend on the onset, compliance, and duration of the treatment. Missing and filled teeth index of patients treated since early childhood is similar to the index of healthy, age-matched controls (64) and dentin mineralization of permanent teeth, which mineralize after birth, can be rescued by the treatment (77) (Figs 4B and 5). The tooth phenotype correlates well with the overall bone phenotype and can be used to evaluate the benefits on mineralization of this treatment (68, 78, 79). Future studies will determine the benefit of treatment on the periodontal status.

### Clinical approaches

Spontaneous dental abscesses can be treated by the conventional endodontic approach, e.g. root canal cleaning to remove the infected pulp tissues and endodontic sealing. However, these abscesses, especially those occurring on primary teeth, spread rapidly in the jawbone and tooth extraction is often necessary. Prevention of abscesses includes sealing the tooth surface (primary and permanent teeth) with a dental resin to form a barrier to bacterial penetration (77). For primary teeth, this non-invasive and painless approach consists in applying an adhesive system (preferably no rinsing step called ‘self-etching’) and then a light-cured flowable resin. This procedure must be repeated regularly (every year) due to gradual wear of the resin until the natural exfoliation of the tooth. In parallel, we recommend rigorous oral hygiene and preventive procedures. Daily use of fluoride toothpaste adapted to age and regular fluoride varnish applications at the dental chair, considering that they present a higher risk of dental infection, is of utmost importance (80). The orthodontic treatment is possible (81), especially in teenagers with HR controlled by the conventional treatment since infancy. In our Reference Center, this treatment is offered to compliant teenagers under conventional treatment when the wave of abscesses occurring in deciduous teeth has ended.

## Novel therapies

In the recent years, novel strategies that efficiently manipulate molecular effectors of the mineral metabolism have been described. Several of them aim at inhibiting downstream FGF23 signaling. Monoclonal FGF23 antibodies with neutralizing effect on FGF23 action have been generated (82). When administered intravenously to *Hyp* mice (murine homolog of XLHR), FGF23 antibodies normalized phosphatemia, increased levels of 1,25-diOH-vitamin D, increased the expression of the Npt2a cotransporter, and tempered the 24-hydroxylase overexpression. Repeated injections to juvenile mice promoted growth and ameliorated mineralization and cartilage development (83). Multiple injections into adult mice were shown to improve spontaneous motor activity as well as muscle strength (84). These promising data prompted the elaboration of a humanized antibody, which showed, in XLH patients, complete absorption and sustained effect on serum phosphate and TmP/GFR beyond 4 weeks upon a single s.c. injection (85). A Phase 2 clinical trial is ongoing in the USA and Canada (86). Two issues may arise with the long-term use of FGF23 antibody. Firstly, it is likely that the anti-FGF23 antibodies will probably not rescue the direct impact of PHEX deficiency on calcified tissue mineralization that depends on MEPE- or OPN-derived ASARM peptides presence in the ECM. Secondly, FGF23 exerts numerous actions including prevention of ectopic calcification through the control of the calcium-phosphate product. Precise modulation of FGF23 signaling will be necessary to avoid off-target actions of FGF23 antibodies.

Another approach was taken by Goetz *et al*. (87); they showed that a C-terminal fragment of FGF23 competes with the full-length protein for receptor binding, yet without activating downstream signaling. Infusion of this FGF23 C-tail in normal rats triggered hyperphosphatemia and renal phosphate retention. In *Hyp* mice, serum phosphate was increased and the fractional excretion of phosphate was decreased compared with control. Despite these promising data, there is no information indicating a possible clinical transition for this elegant approach as of today.

The pharmacological inhibition of downstream FGF23 signaling was also probed using selective pan-specific FGFR antagonists in *Hyp* and *Dmp1*-null mice. Oral administration of the small molecule NVP-BGJ398 led to an improved ion metabolism, restored the structure of the mineralization plate and partly corrected the bone growth impairment (88). Even if the molecule is already in Phase 1 trial in human for cancer therapy, the application to HR will warrant more investigation regarding the selectivity of the molecule, notably towards other FGFR-mediated physiological processes beyond phosphate metabolism.

Noteworthy, parathyroid cells also express the *FGF23* receptor. FGF23 represses PTH secretion, but endocrine feedback loops, while still controversial, have been suggested (89). Normalization of phosphatemia in a TIO patient after parathyroidectomy has been reported recently (90), and manipulation of PTH secretion may therefore help to counteract downstream effects of excessive FGF23 on phosphate and vitamin D metabolism.

In contrast to the above-mentioned strategies that target either FGF23 or its FGFR/KLOTHO co-receptor in the kidney, other efforts are directed toward osteocytic targets. The osteocyte is the primary site of production of *FGF23* and hosts *PHEX* and *DMP1* (malfunctioning in XLHR and ARHR respectively). Osteocytes express the calcitonin receptor and it was recently reported that a single s.c. administration of calcitonin in one patient affected with XLHR results in a decrease in the concentration of *FGF23* and increases in phosphatemia and circulating level of calcitriol (85). A Phase 1 study is currently evaluating repeated daily intranasal calcitonin in XLHR (91).

The identification of a decreased proprotein convertase 2 (PC2) activity in bone cells of *Hyp* mice (92) provides another potential curative option. PC2, and its associated chaperone 7B2, were shown to participate in the proteolytic regulation of the FGF23 protein expression. When hexa-d-arginine (D6R), a PC2 agonist, was administered i.p. into *Hyp* mice, normalization of *7B2* expression and concomitant decrease in *Fgf23* mRNA was observed. Although only partial reduction in the levels of serum *Fgf23* was noted in D6R-treated animals, bone modeling was restored, phosphatemia was sub-normalized, as were the related markers *Npt2a* and *Cyp27b2* encoding the 24-hydroxylase.

Therapies that would be capable of restoring the bone and tooth mineralization process are also desirable. The SIBLINGs phosphoproteins MEPE, DMP1, and OPN all contain ASARM motifs (reviewed in (2)). Upon proteolytic cleavage orchestrated by PHEX, released phosphorylated ASARM peptides interact with hydroxyapatite crystals and inhibit mineralization (93, 94). In XLHR and ARHR, ASARM peptides abundance is increased in the ECM of bone and teeth, and probably contributes to the mineralization defects that hallmark the disease (64, 72). Counteracting ASARM peptides may therefore help in improving bone, dental and periodontal features in XLHR and related diseases.

## Conclusion

Symptoms associated with phosphate wasting result from FGF23 excessive signaling and accumulation of ASARM peptides in bone and dental tissues. Current therapy with phosphate supplements and vitamin D analogs partly correct rickets and osteomalacia. It is obvious that outcomes of therapy are still not optimal and that therapies targeting the pathophysiology of the disease are required. Along this line, FGF23 antibodies are very promising but their benefit remains to be investigated during growth in children with *PHEX,* *FGF23,* or *DMP1* mutation.

Today, and in the future, coordination between caregivers is required for the optimal treatment of this rare disorder and involves various specialists in pediatric and adult fields. While attention is focused in children on legs straightening and growth, adults should be nonetheless regularly monitored for the prevention and limitation of bone pain, joint stiffness, and periodontal inflammation. Extra-osseous issues should be considered early in life, as well as audition, sport practice, and glucose and lipid metabolism, with the help of physiotherapists and nutritionists. In addition, the contribution of psychologist and social workers is necessary, especially in patients born before 1970 who did not receive vitamin D analogs and present multiple and sometime devastating complications. Finally, in the era of globalized communication, active networking involving not only reference centers for rare diseases or scientific societies but also patients organization and biopharmaceutical companies should facilitate the translation of recent discoveries into novel therapies.

## Author contribution statement

All authors contributed equally to this review by writing the text and producing personal data and illustrations; therefore, author's list from 2nd to 11th is by alphabetical order. A Linglart coordinated the review. P Harvengt is the president of the scientific advisory board of the patient's organization, and, as such, represents the patient's association RVRH-XLH.

## Figures and Tables

**Table 1:** Causes of hypophosphatemic rickets (HR).

	**Gene**	**Transmission**	**Reference**	**Main features**
HR sharing elevated FGF23 circulating levels and inappropriately low or normal 1,25-diOH-vitamin D				
XLHR	*PHEX*	X-linked	(9)	HR with similar phenotype in males and females
ADHR	*FGF23*	Autosomal dominant	(10)	HR
ARHR				
	*DMP1*	Autosomal recessive	(11)	HR
ARHR2	*ENPP1*	Autosomal recessive	(12)	HR associated with arterial calcifications of infancy (GACI syndrome)
ARHR3	*FAM20c*	Autosomal recessive	(95)	Hypophosphatemia associated with osteosclerosis of the bone rather than rickets, dysmorphy, and cerebral calcifications; severe dental phenotype
OGD	*FGFR1*		(96)	HR associated with frequent craniosynostosis, dysmorphy, and dwarfism
HR associated with congenital sporadic disorders due to heterozygous post-zygotic mutations in genes activating signaling pathways and elevated FGF23				
MAS			(97)	Characterized by the triad precocious puberty, cafe-au-lait spots and fibrous dysplasia (FD); HR is rare, and secondary to increased FGF23 production by the FD
Mosaic cutaneous disorders include nevus sebaceous and Schimmelpenning syndrome	*KRAS* and *NRAS*		(98)	HR associated with bone lesions and extended cutaneous congenital lesions
HR associated with mesenchymatous tumors secreting FGF23				
TIO			(13)	Acquired and often severe hypophosphatemia and phosphate wasting. Hypocalcemia may be present as the consequence of suppressed 1,25-diOH-vitamin D production
HR sharing appropriately suppressed FGF23 and elevated 1,25-diOH-vitamin D; defects in renal phosphate transporters				
HHRH	*SLC34A3*	Autosomal recessive	(16)	HR with nephrocalcinosis and kidney stones
Diseases affecting the renal distal tubule				
Lowe syndrome, Dent syndrome (CLCN5 gene), Toni-Debré-Fanconi				

XLHR, X-linked HR; ADHR, autosomal dominant HR; ARHR; autosomal recessive HR type 1; ARHR2, autosomal recessive HR type 2; ARHR3, autosomal recessive HR type 3; OGD, osteoglophonic dysplasia; MAS, McCune–Albright syndrome; TIO, tumor-induced osteomalacia; HHRH, hereditary hypophosphatemic rickets with hypercalciuria.

**Table 2:** Objectives and timeline for the conventional treatment of HR in children.

**Interval after start of treatment**	**Objective**
Few weeks	Decrease in bone pain
6–12 months	Normalization of alkaline phosphatase level
1 year	Increase in growth velocity
3–4 years	Straightening of legs: 1 cm decrease in intercondylian (genu varum) or intermalleolar (genu valgum) distance every 6 months

**Table 3:** Reports of vitamin D analogs and phosphate supplements in patients with HR.

**Reference**	**Therapy**	**Subjects**	**Aim of study**	**Main outcomes**
(19)	Phosphate alone 1.2–3.6 g/day in five doses	*n*=11	X-rays and bone histology	Combined phosphate and calcitriol has several advantages over previously described treatment regimens:
	Phosphate in five doses ergocalciferol 25 000–50 000 IU/day	Aged 1.75–11.5 years		Induced mineralization of the growth plate
	Phosphate in five doses calcitriol 1 μg/day			Improved the mineralization of trabecular bone
(99)	Calcitriol mean dose 30 ng/kg per day	*n*=11	Effects of calcitriol on biochemistry and mineralization	Calcitriol raised serum phosphorus in all prepubertal patients but only 2/6 pubertal patients
				No change in renal phosphate threshold
				Improved trabecular bone mineralization
(24)	Calcitriol and phosphorus	*n*=9		Heals rickets
				Changes growth rate
				Decreases alkaline phosphatases
				Symptomatic improvement
(22)	Phase 1	*n*=10	Biochemistry	On calcitriol
	Phosphorus 1.5–3.6 g/day	Age 11.9±2.6 years		Lower PTH levels
	VitD2 10–75 000 U/day			Higher serum phosphorus levels
	Duration 438 months			Lower alkaline phosphatases
	Phase 2			Lower urinary calcium excretion
	Phosphorus 1.5–3.6 g/day			Improved stature
	Calcitriol 17–34 ng/kg per day			
(100)	Calcitriol 58.0±8.5 ng/kg per day in two doses	*n*=19	Growth and mineral metabolism upon switching from ergocalciferol to calcitriol	Improves
	Phosphorus 2167±174 mg/m^2^ per day in five doses			Blood phosphate
				Growth velocity
(101)	Group 1 (1963–1968):	*n*=40	Comparison of three treatment groups and effect on growth and final height	1-αOHD3 promoted catch-up growth and 75% attained normal adult height
	Phosphorus <1 g/day	Aged two and up		Better results than previous treatment regimens
	Cholecalciferol or ergocalciferol 0.5–2 mg/day			
	Group 2 (1968–1978)			
	Phosphorus 0.7–2 g/day			
	25-Hydroxyvitamin D3 or alfacalcidiol 50–200 μg/day			
	Group 3 (after 1978)			
	Phosphorus 0.7–2 g/day			
	Calcitriol 1–3 μg/day			
(21)	Calcitriol 25.6±16.9 ng/kg per day	*n*=24	Comparison to 16 untreated patients (<1971) for height and nephrocalcinosis	Treatment with phosphate and calcidiol increases growth velocity
	Phosphate 100±34 mg/kg per day	Age: 1–16 years (median=5.3 years)		Nephrocalcinosis is a complication of therapy and is associated to the dose of phosphorus (not correlated to the dose of vitamin D analogs or duration of therapy)
(102)	Phosphorus 53–90 mg/kg per day	*n*=13	Identify treatment factors that might be associated to transition of secondary hyperparathyroidism to tertiary hyperparathyroidism	Patients with tertiary hyperparathyroidism (*n*=2) had earlier onset and longer duration of treatment, higher dose of phosphorus and longer duration of treatment with very high phosphorus doses (>100 mg/kg per j) compared with patients with secondary hyperparathyroidism (*n*=11)
	Calcitriol dose 11–27 ng/kg per day			
(17)	Phosphate 80–99 mg/kg per day	*n*=8 aged 0.15–0.58 years		Patients who started the treatment earlier (group 1) had best controlled alkaline phosphatases, a better growth and predicted adult height
	Calcitriol 20 ng/kg per day	*n*=11 aged 1.3–8 years		
		At start of treatment		

**Table 4:** Ranges of doses of phosphate supplements and vitamin D analogs throughout life, and their respective markers of efficacy and safety as applied in our center.

**Period**	**Phosphate supplements**	**Vitamin D analogs** (alfacalcidol only^a^)	**Surveillance for efficacy and safety**	**Frequency**
Infancy (dose) divided into	55–70 mg/kg per day	1.5–2.0 μg/day once/day	Clinical: height, weight, cranial circumference	Every 3 months
	four times/day		Blood: alkaline phosphatases, total calcium, PTH, creatinine	
			Urines (spot): calcium/creatinine	
Childhood (dose) divided into	45–60 mg/kg per day	1.0–2.0 μg/day	Clinical: height, weight, leg bowing, teeth	Every 6 months
	three times/day	once/day	Blood: alkaline phosphatases, total calcium, PTH, creatinine	Every 6 months
			Urines (24-h): calciuria, phosphaturia	Every 3 months
			Renal ultrasound	Every year
Puberty (dose) divided into	35–50 mg/kg per day	1.5–3.0 μg/day	Clinical: height, weight, leg bowing, teeth	Every 6 months
	three times/day	once/day	Blood: alkaline phosphatases, total calcium, PTH, creatinine	Every 6 months
			Urines (24-h): calciuria, phosphaturia	Every 3 months
			Renal ultrasound	Every year
Adulthood (dose) divided into	0–2000 mg/day	0–1.5 μg/day	Clinical: weight, mobility, pain, teeth	Every year
	two times/day	once/day	Blood: bone alkaline phosphatases, total calcium, PTH, creatinine	Every year
			Urines (24-h): calciuria	Every 6 months
			Renal ultrasound	Every other year
Pregnancy (dose) divided into	2000 mg/day	1–1.5 μg/day	Clinical: weight, mobility, pain	Every 3 months
	two times/day	once/day	Blood: total calcium, PTH, creatinine, 25-OH vitamin D	Every 3 months
			Urines (24-h): calciuria	Every 3 months
Menopause (dose) divided into	0–2000 mg/day	0–1.5 μg/day	Clinical: weight, mobility, pain, teeth	Every year
	two times/day	once/day	Blood: bone alkaline phosphatases, total calcium, PTH, creatinine	Every year
			Urines (24-h): calciuria	Every 6 months
			Renal ultrasound	Every other year

a Equivalent dose in calcitriol was obtained divided by a factor 2.
